# The Association between Negative Symptoms, Psychotic Experiences and Later Schizophrenia: A Population-Based Longitudinal Study

**DOI:** 10.1371/journal.pone.0119852

**Published:** 2015-03-06

**Authors:** Nomi Werbeloff, Bruce P. Dohrenwend, Rinat Yoffe, Jim van Os, Michael Davidson, Mark Weiser

**Affiliations:** 1 Department of Psychiatry, Sheba Medical Center, Tel-Hashomer, Israel; 2 Department of Psychiatry and Mailman School of Public Health, Columbia University, New York, New York, United States of America; New York State Psychiatric Institute, New York, New York, United States of America; 3 Division of Mental Health Services, Ministry of Health, Jerusalem, Israel; 4 Department of Psychiatry and Psychology, Maastricht University Medical Centre, Maastricht, The Netherlands; 5 Department of Psychosis Studies, King's College London, King's Health Partners, London, United Kingdom; UTHSCSH, UNITED STATES

## Abstract

**Background:**

Psychotic experiences are common in the general population, and predict later psychotic illness. Much less is known about negative symptoms in the general population.

**Method:**

This study utilized a sample of 4,914 Israel-born individuals aged 25–34 years who were screened for psychopathology in the 1980's. Though not designed to specifically assess negative symptoms, data were available on 9 self-report items representing avolition and social withdrawal, and on 5 interviewer-rated items assessing speech deficits, flat affect and poor hygiene. Psychotic experiences were assessed using the False Beliefs and Perceptions subscale of the Psychiatric Epidemiology Research Interview. Psychiatric hospitalization was ascertained 24 years later using a nation-wide psychiatric hospitalization registry.

**Results:**

After removing subjects with diagnosable psychotic disorders at baseline, 20.2% had at least one negative symptom. Negative symptoms were associated with increased risk of later schizophrenia only in the presence of strong (frequent) psychotic experiences (OR = 13.0, 9% CI: 2.1–79.4).

**Conclusions:**

Negative symptoms are common in the general population, though the majority of people with negative symptoms do not manifest a clinically diagnosed psychiatric disorder. Negative symptoms and psychotic experiences critically depend on each other’s co-occurrence in increasing risk for later schizophrenia.

## Introduction

Numerous studies and meta-analyses have shown that attenuated forms of positive symptoms (psychotic experiences) are not uncommon in the general population [1], and predict later psychotic illness [2–6]. Much less is known about the prevalence of negative symptoms in the population, including poverty of thought and speech, impoverished emotional experience, diminished affect, decreased motivation toward goal-directed behavior, and decreased social drive [7]. The only population-based report on negative symptoms in the general population is a recent longitudinal study by Dominguez and colleagues[8], that found that 15.7% of adolescents and young adults reported lifetime cumulative incidence of negative/disorganized symptoms alone, and 5.5% reported both positive and negative symptoms. Negative symptoms were predictive of positive symptoms over time, and particularly the co-occurrence of positive and negative symptoms was associated with increased probability of functional impairment and help-seeking behavior [8]. However, the association between negative symptoms and hospitalization for schizophrenia has not yet been examined in a population-based sample.

Given the prevalence and functional impact of negative symptoms, it is important to further our knowledge on the prevalence of such symptoms in the general population, and their association with later mental disorders. This study aimed to examine the prevalence of negative symptoms in the general population and their association with psychotic experiences and with later hospitalization for schizophrenia, utilizing data from a population-based cohort of individuals assessed as young adults, and then followed for 24 years.

## Methods

The association between negative symptoms and subsequent psychiatric hospitalization was examined by merging data from an epidemiological study conducted in Israel in the 1980's with data from the National Psychiatric Hospitalization Case Registry. After receiving approval from the IRB at the Sheba Medical Center, these databases were linked in 2007 using the national identification number (analogous to the US Social Security number) as the linking variable. For subjects who participated in the epidemiological study and appeared in the hospitalization registry, dates of hospitalizations and discharge diagnoses were added to the file. Individuals who had a discharge diagnosis of schizophrenia (ICD-10 F20.0-F20.9) at any hospitalization were considered as clinically diagnosed with schizophrenia.

To preserve subjects' confidentiality, the national identification number was removed before the linked file was transferred to the investigators. Hence, patient records were anonymized and de-identified prior to analysis.

### Epidemiological Study

This study utilized data from a two-stage epidemiological study of mental disorders among young adults in a 10-year birth cohort (1949–1958) conducted in Israel in the 1980's [9,10]. As part of the original study’s goal to compare the risk of selected disorders in socio-economically contrasting ethnic groups, a sample of 19,000 Israel-born individuals was drawn from the population registry and screened for father’s continent of origin (North Africa or Europe) and level of education. This was done to balance socioeconomic status within advantaged and disadvantaged ethnic groups by over-sampling disadvantaged subjects from North African origin with high educational level and advantaged subjects of European origin with low educational level. Based on gender, education, ethnicity, and year of birth, 5,200 subjects were selected.

This cohort-based sample was screened and diagnosed for psychotic and other psychiatric disorders in two stages. During the screening stage, 94.5% (n = 4,914) were interviewed using a Hebrew version of the Psychiatric Epidemiology Research Interview (PERI), a well validated screening tool [11–13]. Data were not obtained for 5.5% subjects (n = 286). Mean age at the time of the interview was 29.4 years (SD = 3.1 years); 49.6% were men. The PERI interview also included self-report of lifetime use of cannabis (including single or rare usage), coded 0 = no, 1 = yes.

Subjects reaching predetermined cut-off values on the PERI screening stage [13], unable to complete the screening interview, or suspected of having a history of psychiatric illness, were referred for a clinical diagnostic (second) stage (n = 2,643). In addition, to determine the rates of false negatives, 18.3% of the 2271 screened negatives—who did not reach the predetermined cut-off values on the PERI—were randomly selected and also referred for a diagnostic interview [10]. From this sub-sample, 2,741 (90.8%) persons were interviewed by psychiatrists using a modified lifetime version of the Schedule for Affective Disorders and Schizophrenia (SADS-I; I for Israel) to arrive at a Research Diagnostic Criteria (RDC) [14] diagnosis. The SADS-I also included an assessment of the best level of social functioning during the five years preceding the interview, rated on a scale of 1 (superior) to 7 (grossly inadequate). Mean time from the screening stage (PERI) to the second stage (SADS-I) was 1.1 years ±11.5 months (range 0–4.5 years).

### Assessment of Negative Symptoms

Two of the study researchers (MW and NW) reviewed the symptoms covered in the PERI and identified items assessing negative symptoms similar to those assessed in the Positive and Negative Syndrome Scale (PANSS) [15] and the Scale for the Assessment of Negative Symptoms (SANS) [16], scales commonly used for the assessment of negative symptoms. Fourteen symptoms (9 self-reported and 5 interviewer-rated; see S1 Table) were identified. The self-report items were scored on a 5-point Likert scale, ranging from (0) “never” to (4) “very often” or “not at all like you” (0) to “very much like you” (4), whereas the interviewer-rated items were scored “present” or absent”.

The fourteen negative symptoms were included in a principal component analysis with varimax rotation. Four symptom clusters were extracted in this analysis: avolition (4 items), social withdrawal (5 items), speech deficits (2 items), flat affect and poor hygiene (3 items) (Table 1). As flat affect and poor hygiene are two distinct concepts, this cluster was split into two: flat affect (2 items) and poor hygiene (1 item).

**Table 1:** Principal component analysis of negative symptoms—item loadings.

	Factor 1	Factor 2	Factor 3	Factor 4
Oversleeping	**0.61**	0.05	−0.04	0.05
Inability to get things done	**0.77**	0.09	0.04	0.01
Trouble getting things started	**0.78**	0.11	−0.01	−0.04
Anergia	**0.53**	0.15	0.08	0.02
Being a loner	0.15	**0.52**	−0.05	0.02
Being a closed person	0.10	**0.78**	−0.03	0.01
Rarely visiting or talking to others	0.12	**0.79**	0.02	0.01
Not making friends easily	−0.01	**0.67**	0.15	0.06
Feeling detached from others	0.37	**0.53**	0.04	0.03
Unclear speech	−0.01	0.06	**0.75**	−0.03
Repeats words mechanically	−0.01	0.01	**0.70**	−0.02
Poor cleanliness and self-care	0.07	0.03	**0.40**	0.17
No external expression	0.05	0.04	0.19	**0.80**
Frozen expression	−0.01	0.06	−0.04	**0.84**

### Assessment of Psychotic Experiences

Self-reports of psychotic experiences were assessed using the 13-item False Beliefs and Perceptions sub-scale of the PERI. Subjects were asked whether they had experienced psychotic experiences and the frequency of each symptom during the past year was recorded on a 5-item Likert scale rated (0) “never”, (1) “rarely”, (2) “sometimes”, (3) “often”, and (4) “very often”. As in previous studies, including one using this sample [5,6,17,18], separate scores were defined for “weak” (occurring rarely or sometimes) and “strong” (occurring often or very often) psychotic experiences. The scale includes questions assessing core positive symptoms such as delusions, hallucinations, bizarre thinking; feeling possessed; feeling dissolved; feeling that thoughts were not one's own; and beliefs of mind control. An example of a typical question is: “have you felt that your mind was dominated by external forces that you had no control over?” The PERI-False Beliefs and Perceptions sub-scale is based on similar criteria to that of the CIDI psychosis section (version 1.1), the favored instrument in similar studies [3,17,19,20]. The PERI screening scales, including the False Beliefs and Perceptions sub-scale, were tested for reliability and criterion validity, and calibrated against known cases of psychiatric disorders in a pilot research project conducted in Israel [13]. In addition to the ability to discern between psychiatric cases and non-cases [13], the False Beliefs and Perceptions sub-scale has high internal reliability (α = 0.80 in the current study and 0.85 in previous studies [21]).

### Psychiatric Hospitalisation Case Registry

The National Psychiatric Hospitalization Case Registry [22,23] is a complete listing of all psychiatric hospitalizations in the country, including psychiatric hospitals, day hospitals, and psychiatric units in general hospitals, using ICD discharge diagnoses assigned by a board-certified psychiatrist. Reporting is regularly monitored to ensure accuracy. A study comparing RDC diagnoses with registry diagnoses found that the sensitivity of the registry diagnosis of non-affective psychotic disorders was 0.89, and of schizophrenia was 0.87 [22,23]. A recent study on this data set showed that 93% of patients with schizophrenia were hospitalized at some point during their lifetime [24].

### Study Population

**Fig 1:**
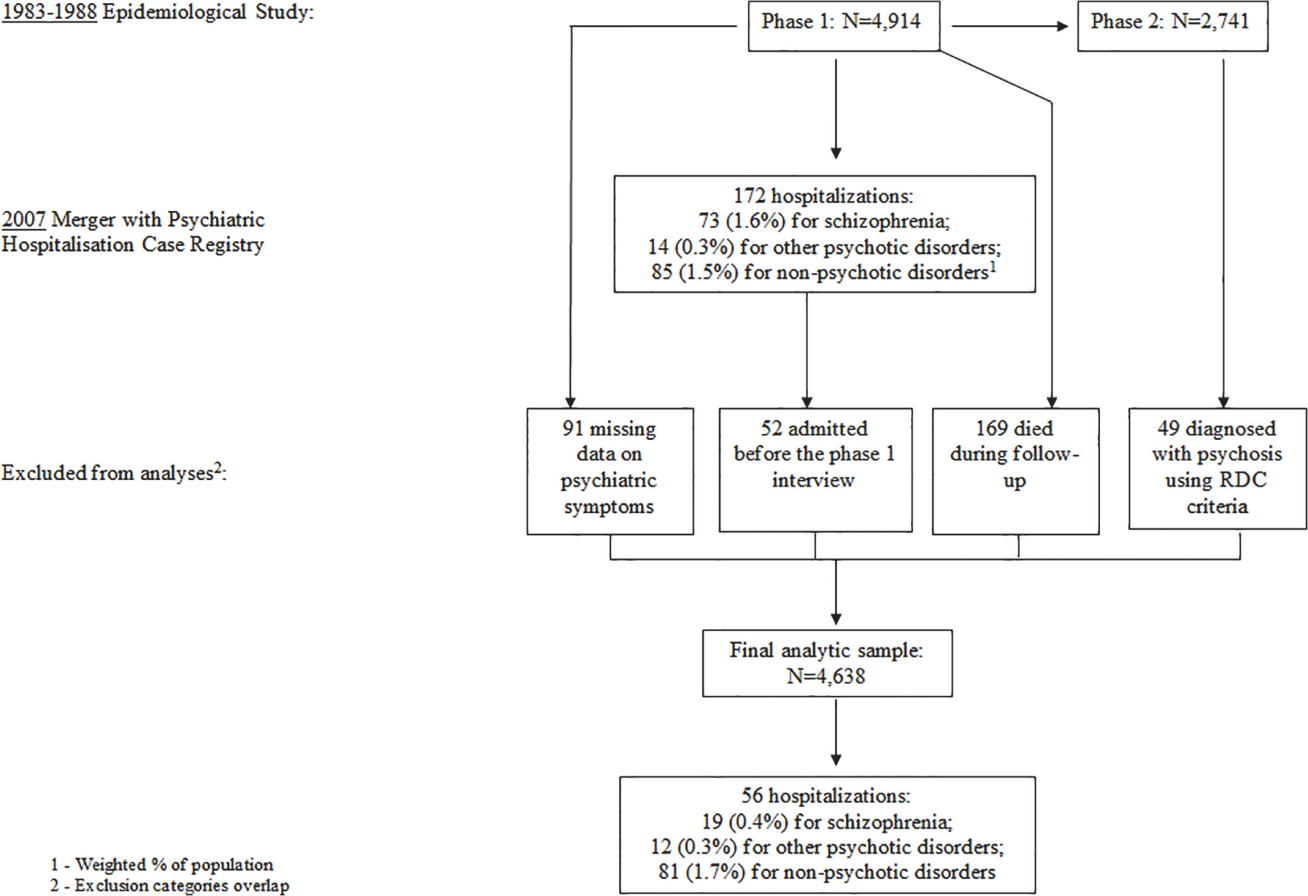
Study population.

Of the 4,914 subjects who participated in the screening interview, 172 had a psychiatric hospitalization, 73 (1.6%) of whom were hospitalized at least once with a diagnosis of schizophrenia either before or after screening. During the follow up, 169 cohort members had died and were thus excluded from the analysis. In addition, we removed from the analyses subjects 1) diagnosed with a psychotic disorder during the SADS-I interview; 2) hospitalized in psychiatric facilities prior to the PERI assessment; and 3) with missing data on the PERI assessment of psychiatric symptoms. The final analytic sample included 4,638 subjects (94.4% of the original sample; see Fig. 1).

During the follow-up period (mean time from PERI interview to first hospitalization or to date of merger in 2007 was 23.5 years, SD = 3.7), 19 individuals (0.4%) were identified in the Psychiatric Hospitalization Case Registry as having been later hospitalized at least once for schizophrenia. The reason for this relatively low rate of schizophrenia (0.4%) in the analytic sample is because the peak incidence of schizophrenia is in the decade 15–24 [25]; thus, the majority of the subjects with this disorder had already manifested their illness at the time of the epidemiological study, and hence they were excluded from this analysis. Twelve subjects (0.3%) were identified as having psychotic disorders other than schizophrenia. An additional 81 subjects (1.7%) were identified as having been hospitalized for non-psychotic disorders, including drug use, personality disorders, affective disorders, anxiety disorders and organic mental disorders.

### Statistical Analysis

All analyses were performed using the software package Stata, version 10 [26]. The data were weighted to estimate the rates of negative symptoms in the original population from which the cohort sample was drawn. This was done using Stata’s tools for complex survey data analysis which incorporates the survey design characteristics (weighting, clustering, and stratification) into the data analysis to provide accurate point estimates and standard errors [27]. All numbers shown represent the actual number of subjects, whereas the percentages are weighted.

As the PERI was not designed to assess negative symptoms, we tested the construct validity of the negative symptoms identified by comparing the prevalence of these symptoms between people who were diagnosed with schizophrenia at the initial assessment in the epidemiological study in the 1980's (n = 24), those diagnosed with other definite RDC mental disorders (n = 1,624) and the rest of the sample.

First, in order to assess the effect of baseline reports of negative symptom on later hospitalization for schizophrenia, with no assumptions regarding the relative importance of the frequency at which the symptom was endorsed, we created for each subject a mean negative symptom score of the 9 self-report items (rated 0–4). This mean score was then used in a logistic regression model to predict later hospitalization for schizophrenia.

Next, for purposes of analysis, each self-report item was dichotomized as present (indicating a score of 4) or absent (indicating a score of 0–3), similar to the coding of the interviewer-rated items. We then examined the prevalence of negative symptoms in the population, as well as the prevalence of negative symptom clusters (avolition, social withdrawal, speech deficit, flat affect and poor hygiene). Next, a logistic regression analysis examining the presence of any negative symptom and risk of later hospitalization for schizophrenia was performed. To examine the specificity of negative symptoms as predictors of schizophrenia, risk of hospitalization for non-psychotic disorders was also tested. For all logistic regression models, the reference group was people who did not report any negative symptoms. Associations were expressed as odds ratios and 95% confidence intervals (95% CI).

Finally, we divided those with negative symptoms into sub-groups based on the co-occurrence of psychotic experiences (i.e, negative symptoms alone or negative symptoms and psychotic experiences together). We then estimated the interaction between psychotic experiences (coded as absent / weak / strong) and negative symptoms (coded as absent / present) in the model predicting hospitalization for schizophrenia, comparing multiplicative effect sizes (odds ratios) using the Stata test command.

## Results

### Construct validity of negative symptom scale

The construct validity of the negative symptom scale was examined by comparing the prevalence of these 14 symptoms between 24 subjects diagnosed with schizophrenia at the initial assessment in the epidemiological study, those diagnosed with other definite RDC mental disorders and the rest of the sample. As can be seen in Table 2, the majority of the PERI items hypothesized to reflect negative symptoms were more prevalent among people diagnosed with schizophrenia. The distinction between subjects with schizophrenia and the general population was non-significant for three items: “rarely visiting or talking to others”, “not making friends easily”; and interviewer rating of “frozen expression”. Of the 14 symptoms assessed, six were significantly more prevalent in subjects with schizophrenia than in subjects with other mental disorders and 2 additional items showed a trend in the same direction. The prevalence of “rarely visiting or talking to others” was numerically higher among subjects with other mental disorders than among subjects with schizophrenia, though both groups differed significantly from the general population.

**Table 2:** Construct validity of negative symptoms—comparison of prevalence (χ^2^) between people with a RDC diagnosis of schizophrenia or other mental disorders and the general population*.

	symptom	Prevalence			PSZ vs. general population	PSZ vs. other disorders
		General population(n = 3,241)	Other definite mental disorders(n = 1,624)	Schizophrenia(n = 24)		
Self-reported	Oversleeping	1.6%	6.8%	45.7%	<.001	<.001
	Inability to get things done	0.4%	2.9%	6.8%	<.001	.23
	Trouble getting things started	0.3%	3.6%	54.1%	<.001	<.001
	Anergia	0.9%	2.1%	13.7%	<.001	.001
	Being a loner	0.6%	3.4%	11.3%	<.001	.07
	Being a closed person	0.5%	3.7%	8.6%	<.001	.24
	Rarely visiting or talking to others	0.9%	5.5%	3.1%	.12	.45
	Not making friends easily	2.8%	4.3%	3.1%	.90	.68
	Feeling detached from others	0.3%	1.7%	7.4%	<.001	.04
Interviewer-rated	Unclear speech	0.7%	2.4%	4.7%	.01	.42
	Repeats words mechanically	0.6%	0.7%	10.3%	<.001	<.001
	No external expression	3.1%	4.4%	12.5%	.01	.07
	Frozen expression	2.7%	3.0%	4.9%	.48	.54
	Poor cleanliness and self-care	2.3%	4.0%	30.0%	<.001	<.001

* Numbers in the table represent the actual number of subjects; percentages represent weighted % among each diagnostic group (column)

### Prevalence of negative symptoms and association with baseline demographics and some clinical characteristics

The weighted mean age of the sample in 2007 was 53.7 years (SD = 2.8), 50% were male. After removing subjects with diagnosable psychotic disorders at baseline, 20.2% (n = 1,054) had at least one negative symptom. Of these, 1.6% (n = 9) were later hospitalized for schizophrenia, 0.1% (n = 1) were hospitalized for psychotic disorders other than schizophrenia, and 3.4% (n = 38) were hospitalized for other psychiatric disorders. A closer inspection of the symptom clusters suggests that 8.3% of the respondents endorsed symptoms of social withdrawal, 6.5% avolition, 5.7% flat affect, 2.8% poor hygiene and 1.7% exhibited speech deficits.

Table 3 shows that negative symptoms (one or more) were more prevalent among males, subjects from North African origin, and those who were unmarried, unemployed and with fewer years of education. Similarly, the prevalence of negative symptoms was higher among cannabis users and participants with poorer social functioning. The prevalence of negative symptoms was 1.5 times higher among those with positive psychotic experiences compared to those without psychotic experiences (Table 3).

**Table 3:** The prevalence of at least one negative symptoms by demographic and clinical characteristics (χ^2^)*.

		Prevalence of negative symptoms	P-value
Sex	Male	572 (23.5%)	<.001
	Female	482 (17.0%)	
Ethnicity	European	377 (15.8%)	<.001
	North African	677 (29.5%)	
Marital status	Married	708 (17.9%)	<.001
	Unmarried	346 (31.8%)	
Education	<12	590 (34.2%)	<.001
	> = 12	459 (14.2%)	
Employment	Employed	940 (19.5%)	<.001
	Unemployed	114 (32.2%)	
Cannabis use	No	852 (19.1%)	<.001
	Yes	202 (27.9%)	
Social functioning	Good-superior	448 (20.8%)	<.001
	Poor-fair	208 (46.0%)	
Positive psychotic experiences (weak or strong)	Absent	205 (15.1%)	<.001
	Present	849 (22.3%)	

* Numbers in the table represent the actual number of subjects; percentages represent the weighted % among each level of the variable examined (row).

### Prediction of hospitalization for schizophrenia (ICD-10 F20.0-F20.9)

To assess the effect of self-report negative symptoms on later hospitalization for schizophrenia, with no assumptions regarding the way in which negative symptoms should be modeled, we examined the association using the mean negative symptom scores. Higher negative symptom scores significantly increased the risk of later hospitalization for schizophrenia (OR = 2.80, 95% CI: 1.67–4.68), indicating that with every unit increase in the mean negative symptom score there was almost a three-fold increase in risk of later hospitalization for schizophrenia.

Overall, when examining all 14 dichotomized negative symptoms, the presence of any negative symptom was associated with increased risk for later hospitalization with an ICD-10 diagnosis of schizophrenia (OR = 9.0, 95% CI: 2.6–30.4). However, when examining the interaction between psychotic experiences and negative symptoms on risk of hospitalization for schizophrenia (Table 4), the significant increase in risk was limited only to the co-occurrence of both strong positive psychotic experiences and negative symptoms; here the risk for later schizophrenia was greatest, at OR = 13.0 (95% CI: 2.1–79.4). The results of the interaction analyses indicate that a trend towards significance was observed for both weak [F(1, 4544) = 3.03, p = .08] and strong psychotic experiences [F(1, 4544) = 2.66, p = .10]. Results of classic regression models including main effects and interaction terms yielded similar results (negative symptoms and weak psychotic experiences: OR = 14.7, p = .08; negative symptoms and strong psychotic experiences: OR = 11.3, p = .10). While these analyses were underpowered to detect significant differences, the results suggest that risk of hospitalization for schizophrenia given both psychotic experiences (weak or strong) and negative symptoms was greater than the sum of the risks for schizophrenia given psychotic experiences or negative symptoms alone.

**Table 4:** Interaction between psychotic experiences and negative symptoms on risk of hospitalization for schizophrenia*.

	No hospitalization(n = 4,526)	Schizophrenia hospitalization(n = 19)	Risk for schizophrenia hospitalizationOR (95% CI)
None	985 (99.8%)	3 (0.02%)	1
Weak psychotic experiences	2,106 (99.9%)	4 (0.01%)	0.6 (0.1–3.3)
Strong psychotic experiences	431 (99.7%)	3 (0.03%)	1.5 (0.3–8.1)
Negative symptoms	198 (99.8%)	1 (0.02%)	0.8 (0.1–8.0)
Weak psychotic experiences and negative symptoms	478 (98.4%)	3 (1.6%)	7.0 (0.9–52.6)
Strong psychotic experiences and negative symptoms	328 (97.1%)	5 (2.9%)	13.0 (2.1–79.4)

* Numbers in the table represent the actual number of subjects; percentages represent the weighted % among each symptom group (row)

### Prediction of hospitalization for other, non-psychotic psychiatric disorders

Higher mean negative symptom scores significantly increased the risk of later hospitalization for non-psychotic disorders (OR = 1.74, 95% CI: 1.09–2.77), though to a lesser degree than risk for schizophrenia. Similarly, the presence of any negative symptom was associated with increased risk of hospitalization for other, non-psychotic psychiatric disorders, though to a lesser degree (OR = 3.5, 95% CI: 2.0–6.0). No interaction was found between negative symptoms and weak [F(1, 4606) = 1.49, p = .23] or strong psychotic experiences [F(1, 4606) = 1.87, p = .17] on risk of hospitalization for non-psychotic disorders.

## Discussion

Similar to psychotic experiences, negative symptoms are common in the general population. The prevalence found in this study is similar to that reported in a population-based study in Germany and in a study of American college students [28], finding that between 20–22% of the general population had one or more negative symptoms. While negative symptoms are common in the general population, the vast majority of people with negative symptoms do not manifest a clinically diagnosed psychiatric disorder. This report adds to the existing literature as it is the first to examine the association between negative symptoms in the general population and hospital diagnoses of schizophrenia in a population-based sample, is based on a birth cohort and has the longest duration of follow-up.

Negative symptoms were more prevalent among males, subjects from North African origin, and those who were unmarried, unemployed and with fewer years of education. The prevalence of negative symptoms was higher among cannabis users and participants with poorer social functioning. This indicates that negative symptoms share some demographic patterns and risk factors with schizophrenia [29,30].

Overall, negative symptoms appeared to be associated with both later schizophrenia and, to a lesser degree, non-psychotic disorders. However, when examining the interaction between psychotic experiences and negative symptoms, the findings indicated that neither negative symptoms nor psychotic experiences alone predicted later schizophrenia. It was only the combined presence of both strong positive psychotic experiences and negative symptoms together that showed a statistically significant risk for later schizophrenia. Although the data in this report cannot speak to this possibility, it supports the notion presented by Moller [31] that in the prodromal phase of the illness, negative symptoms begin worsening with the emergence of positive symptoms, leading to the initial acute psychotic episode. This was later confirmed in a longitudinal study on the topic reporting that transition from psychotic experiences to psychotic disorder is contingent on the earlier presence of negative symptoms [8].

### Limitations

The assessment of negative symptoms in this study was done using items that were not designed as a scale for the assessment of negative symptoms, and were not previously validated for this purpose. However, the present data analysis suggests that the symptoms examined were all more prevalent in people with schizophrenia, hence supporting the construct validity of most of these items. Additionally, not all negative symptoms were evaluated. For example, anhedonia or lack of emotional experience was not assessed as part of the epidemiological survey. Another potential limitation is that 9 of the 14 negative symptoms were based on self-report rather than on clinical observations.

The diagnoses of schizophrenia were drawn from a psychiatric hospitalization registry. Registry diagnoses are clinical, not research, diagnoses. However, they are assigned by board-certified psychiatrists who had the benefit of observing the patients throughout one or more hospitalizations and had been trained in the use of the diagnostic criteria of the ICD-10 and DSM-IV. Studies that compared clinical diagnoses of schizophrenia assigned in state hospitals with research diagnoses have shown a high degree of concordance [32], and a previous study by our group comparing registry diagnoses to RDC diagnoses found that the sensitivity of the registry diagnosis of non-affective psychotic disorders is 0.89, and of schizophrenia 0.87 [22].

We are also limited by lack of data regarding emigrants. However, recent studies on schizophrenia and emigration argue against the possibility that patients more prone to schizophrenia are more likely to emigrate compared to those less prone to schizophrenia [33].

Finally, after removing subjects with diagnosable psychotic disorders at baseline, only 19 people with schizophrenia, the main outcome of interest, were included in the analytic sample. Hence, the regression models may be underpowered, resulting in large confidence intervals. For this reason, the inclusion of potential covariates in the models was not possible. As people whose psychotic illness had manifested prior to the baseline assessment (ages 25–34) were removed from the analyses, and as the peak incidence for males and females is in the decade 15–24 [25], the remaining participants are characterized by a later age of onset, which may limit the generalizability of these findings.

## Supporting Information

S1 Table Assessment of negative symptoms. (DOCX)
