# Supplementary material for: The Association between Negative Symptoms, Psychotic Experiences and Later Schizophrenia: A Population-Based Longitudinal Study
Source: PLoS One. 2015 Mar 6;10(3):e0119852. doi: 10.1371/journal.pone.0119852 (PMC4351950; doi:10.1371/journal.pone.0119852)
Supplement: S1 Table — (DOCX) [file pone.0119852.s001.docx]

**Table S1. Assessment of negative symptoms**

|  | Symptom |  |  |
| --- | --- | --- | --- |
|  |  | Original question | Scoring scale |
| Self-reported | Oversleeping | How often in the past year have you slept past the time you wanted to get up? | Never / almost never / sometimes / fairly often / very often |
|  | Inability to get things done | How often in the past year have you felt unable to get things done? | Never / almost never / sometimes / fairly often / very often |
|  | Trouble getting things started | How often in the past year have you had trouble getting started on the things you have to do? | Never / almost never / sometimes / fairly often / very often |
|  | Anergia | How often in the past year have you felt full of energy?* | Never / almost never / sometimes / fairly often / very often |
|  | Being a loner | Think of a person who is a known loner (likes to be alone). Is this person… | Not at all like you / very little like you / somewhat like you / much like you / very much like you |
|  | Being a closed person | Think of a person who people say is closed in himself. Is this person… | Not at all like you / very little like you / somewhat like you / much like you / very much like you |
|  | Rarely visiting or talking to others | Think of a person who rarely visits people or who doesn't call them by phone. Is this person… | Not at all like you / very little like you / somewhat like you / much like you / very much like you |
|  | Not making friends easily | Thinks of a person who makes friends easily. Is this person…* | Not at all like you / very little like you / somewhat like you / much like you / very much like you |
|  | Feeling detached from others | How often in the past year have you felt detached from other people? | Never / almost never / sometimes / fairly often / very often |
| Interviewer-rated | Unclear speech | Interviewee's speech was unclear | Yes / no |
|  | Repeats words mechanically | Interviewee repeats words and sentences over and over in a mechanical way | Yes / no |
|  | No external expression | Interviewee talks about problems with no external expression of feelings | Yes / no |
|  | Frozen expression | Interviewee keeps a frozen expression without any signs of emotion | Yes / no |
|  | Poor cleanliness and self-care | Interviewee’s cleanliness and self-care | Clean / not clean |

*Item reversed for purposes of analyses.
